# T3DB: an integrated database for bacterial type III secretion system

**DOI:** 10.1186/1471-2105-13-66

**Published:** 2012-04-30

**Authors:** Yejun Wang, He Huang, Ming’an Sun, Qing Zhang, Dianjing Guo

**Affiliations:** 1 School of Life Science, The Chinese University of Hong Kong, Shatin, NT, Hong Kong; 2 Institute of Pathogen Biology, Chinese Academy of Medical Sciences and Peking Union Medical College, Beijing, China

## Abstract

**Background:**

Type III Secretion System (T3SS), which plays important roles in pathogenesis or symbiosis, is widely expressed in a variety of gram negative bacteria. However, lack of unique nomenclature for T3SS genes has hindered T3SS related research. It is necessary to set up a knowledgebase integrating T3SS-related research data to facilitate the communication between different research groups interested in different bacteria.

**Description:**

A T3SS-related Database (T3DB) was developed. T3DB serves as an integrated platform for sequence collection, function annotation, and ortholog classification for T3SS related apparatus, effector, chaperone and regulatory genes. The collection of T3SS-containing bacteria, T3SS-related genes, function annotation, and the ortholog information were all manually curated from literature. BPBAac, a highly efficient T3SS effector prediction tool, was also implemented.

**Conclusions:**

T3DB is the first systematic platform integrating well-annotated T3SS-related gene and protein information to facilitate T3SS and bacterial pathogenecity related research. The newly constructed T3 ortholog clusters may faciliate effective communication between different research groups and will promote *de novo* discoveries. Besides, the manually-curated high-quality effector and chaperone data are useful for feature analysis and evolutionary studies of these important proteins.

## Background

Type III Secretion System (T3SS) is a complex protein secretion system that plays pivotal roles in bacterial pathogenesis. T3SS is widely distributed in different gram negative bacteria [1,2]. Bacteria containing functional T3SSs can invade animal or plant hosts, causing different human, animal and plant diseases, such as human or animal plague, typhoid, dysentery, cholera and enteritis, and plant wilt, blight, cankers and leaf spots [1,2]. In addition, recent studies suggest that T3SSs also function in the symbiosis between different symbiotic bacteria and their eukaryotic hosts [3,4]. One bacterium may contain more than one T3SS [5,6].

Although T3SSs have been identified in many bacterial species, quite limited number of T3SSs has been extensively investigated. Till now, 5 animal pathogens (*SalmonellaShigellaYersinia*, pathogenic *E. coli* and *Citrobacter*) and 2 plant pathogens (*Pseudomonas* and *Xanthomonas*) have been well studied for their T3SSs [7-12]. The relatively low sequence similarity and frequent horizontal transfer among bacteria makes it difficult to identify T3SS orthologs [13,14]. Most importantly, different nomenclature and categorization methods/terms have created confusion and difficulty in literature search as well as in data interpretation using relevant information obtained in other genera [13,14]. A unified platform integrating various sources of information is urgently needed to facilitate T3SS related research.

Pallen MJ et.al. established a database (http://3base.bham.ac.uk) aiming to formulate a taxonomy for type-III secretion, and to facilitate T3SS identification in newly-sequenced bacterial genomes [13]. However, the database so far only contains annotated T3SSs from 4 species. Other databases, such as T3SEdb and Effective, mainly store subsets of validated and predicted T3SS effectors [15,16]. A systematic function annotation of individual T3SS effector coding genes, the relationship between these effectors and their chaperones, the structural components and the regulators of corresponding T3SSs may provide useful multiple-aspect knowledge for further studies on T3SS-related gene evolution, chaperone-effector interaction, regulatory network, and bacteria-host interaction, etc. Regretfully, no such information can be found in any of the current databases. In addition, none of the current platforms implements various computational software tools for type 3 effector prediction.

In this study, we developed the T3SS-related Database (T3DB), in which we annotated and categorized T3SS-related genes into 4 major categories: apparatus, chaperones, effectors and regulators. For each gene, its genome coordinate, gene accession, gene alias, nucleic acid sequence, protein sequence, structure information, and detailed function were annotated. All these data were manually annotated, and experimental evidence was provided for each effector, chaperone or transcription regulator. A term, ‘T3 orthologs’, was invented to describe evolutionarily and functionally related T3SS genes. T3 ortholog clusters and effector-chaperone pairs can be freely downloaded. Besides, BPBAac, an efficient T3SS effector prediction tool was implemented in a web server [17]. The links to other T3SS effector prediction servers are also provided [18-20].

## Construction and content

**Figure 1:**
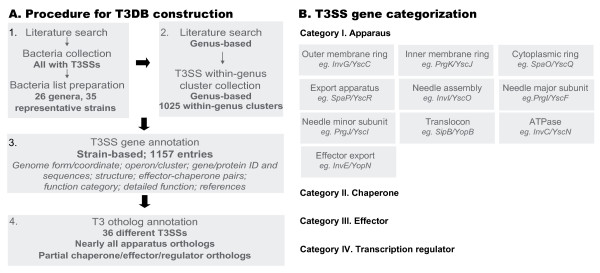
**Construction of T3DB and T3SS gene categorization.** Construction of T3DB involved 4 steps. The annotation work and main achievements for each step are shown in (A). (B) lists the major T3SS gene categories annotated in T3DB. For each subgroup of Apparatus, examples are given from *Salmonella* SPI-1 and *Yersinia* Ysc T3SSs.

Figure 1A demonstrated the framework for T3DB construction, which involves 4 steps: (1) identification of T3SS containing bacterial genera and species; (2) T3SS gene identification and categorization; (3) T3SS gene annotation; (4) ortholog annotation.

First, a text based literature search strategy was adopted to obtain a comprehensive list of T3SS related publications. ‘Type III Secretion System’, ‘Type 3 Secretion System’, ‘TTSS’ and ‘T3SS’, were respectively used as key words to search the PubMed database. This search resulted in more than 3000 non-redundant publications. The abstract of each publication was scanned manually, and the bacterial genera and species were recorded and examined. Because some bacteria may contain not yet reported candidate T3SSs, instead of using comprehensive sequence alignments to find these candidates, we only included potential T3SS candidates based on literature reviews in which the authors presented sequence alignments, genome localization, and phylogenetic evidence.This procedure generated a list of 26 bacterial genera from different classes, even from different phyla (http://biocomputer.bio.cuhk.edu.hk/T3DB/browse). The phylogenetic relationship between these bacterial genera was annotated from Bergey’s Manual [21]. For each genus, the model species and strains with the most adequate experimental data and molecular information were further selected. The genomes (chromosomes and plasmids) of most of the selected model strains have been sequenced, and the current release contains 35 model species. The host type (animal or plant) and interaction type (pathogenesis or symbiosis) were annotated for each species according to Bergey’s Manual [21].

All the T3SS-related genes were then collected for each selected model strain. Due to the lack of high sequence similarity among different genera, especially among distantly-related genera, it is difficult to identify T3SS-related genes only based on sequence alignment using fixed parameters and reference gene sets. Accordingly, a manual curation from literature search was combined. Genes that have been reported to relate with functional T3SSs (e.g., being secreted or translocated through T3SSs, regulating putative T3SS genes, or assisting secretion of T3SS effectors), or have sequence or function conservation with T3SS genes in other bacterial species/genera were retrieved. Because it is much easier to identify orthologs in bacteria that belong to the same genus, each genus was therefore used as key word in combination with either ‘Type III Secretion System’, ‘Type 3 Secretion System’, ‘TTSS’, or ‘T3SS’ to search relevant literature in the PubMed database. Each literature hit was manually curated and genes related to T3SS were collected, together with their bacterial host strain, alias, gene accession, and detailed function. Furthermore, the candidate gene sequences and their genomic coordinates were tracked and compared, and T3SS orthologs in different species or strains were identified. Because a strain may contain more than one T3SS, and genes with similar sequence, structural, function and genomic clustering features among different T3SSs in the same strain can not be accurately defined as paralogs or orthologs, we created a new term ‘T3 ortholog’ to specify this case. Specifically, any genes with the above-stated features among different T3SSs in the same or different strains were collectively termed ‘T3 orthologs’. A non-redundant T3 ortholog cluster set was obtained for each genus after clustering the within-genus and inter-T3SS T3 orthologs. Each gene cluster in the genus-based non-redundant T3 ortholog cluster set was assigned a unique name, in the form of ‘XXX-YYY’, where ‘YYY’ is the traditional gene name for that gene in most studied strains and ‘XXX’ describes the genus. The genus name was included in the gene name so that users can easily distinguish the genus from which the candidate gene originates. Even in the same genus, the orthologs in different strains may have different names. After a unique nomenclature was set for each ortholog cluster in a genus, other names representing the same gene were considered as aliases. For strains with more than one T3SS, the nomenclature for genes was in the format of ‘XXX-ZZZ-YYY’, where ‘XXX’ and ‘YYY’ denote genus and gene name respectively, and ‘ZZZ’ describes the T3SS name. Each T3SS was classified into one of the five putative categories [22] according to phylogenetic analysis of the conserved T3SS proteins among all bacteria that contain T3SSs.

After the within-genus T3 ortholog clusters were set up for T3SSs in each genus, most genes in T3 ortholog clusters could be tracked directly from literature for most representative strains (Case I). For other strains, however, many T3SS related genes were not well annotated (Case II) and we could not track the T3 ortholog clusters directly from literature according to the function annotation. Therefore, a comparative genomic analysis was further conducted between different representative bacterial strains, combined with sequence alignment. If a Case II gene in strain A and its two flanking genes had the same gene order with a Case I gene and its flanking genes in strain B, and two corresponding genes had a >90% amino acid identity,the Case II gene in strain A was considered as a putative T3SS-related gene, and was classified into the corresponding T3 ortholog cluster. Due to evolutionary gene loss, pseudogenes, sequencing errors, and large sequence divergence, after this annotation, many strains still contain only a subset of the within-genus T3 ortholog genes.

The T3SS genes annotated in T3DB are divided into 4 major categories (Figure 1B): apparatus (Category I), chaperone (Category II), effector (Category III), and transcription regulator (Category IV). Apparatus genes encode those that assemble the needle-like structure as well as accessory genes. Genes in this category are further sub-classified into different function clusters (Figure 1B). Chaperone genes encode proteins that assist effector proteins to secrete through T3SS conduit. Effectors genes encode proteins specifically secreted through T3SS conduit. Some effectors themselves also function as structure proteins, such as those translocon proteins (e.g., Sal-SPI1-SipB and SipC). In such cases, they were classified into ‘Translocon’ in Category I. T3SS transcription regulators were collected as an independent category. For categories chaperone, effector and transcription regulator, at least one reference with experimental evidence was required to support the function annotation. For apparatus (Category I), sequence similarity and genomic organization were used as evidence, for which two conditions must be both met. For bacteria that contain multiple-T3SSs, some effectors cannot be precisely classified to a specified T3SS; in such case, the name of the orthologous gene cluster adopts ‘XXX-YYY’ instead of ‘XXX-ZZZ-YYY’ system.

For gene annotation, orthologous genes in different strains within the same genus adopt the same gene names. To distinguish these genes from different strains, a unique ID was assigned to each gene. The ID is represented by T3X, where ‘X’ is one of the four characters (‘A’: Apparatus; ‘C’: Chaperone; ‘E’: Effector; ‘R’: Regulator), followed by 11 numerical numbers representing different phyla (1 number), classes (1 number), orders (1 number), families (1 number), genera (2 numbers), strains (2 numbers), T3SSs in the same strain (1 number), and the individual genes (2 numbers), respectively. It should be noted that when more than one T3SSs are presented in a single strain and one is not able to determine to which T3SS the gene belong, the corresponding number is replaced by a character ‘x’. For each gene, the genome type (chromosome or plasmid), genome ID and gene coordinates in genome (if available), strand direction, nucleic acid and protein sequences, major function category, detailed function annotation, structure information, and reference PMIDs were all annotated.

In the last step, an inter-genus ‘T3 ortholog’ cluster was annotated for each gene. As defined in the previous paragraphs, the term ‘T3 orthologs’ was proposed because wide horizontal gene transfer events has led to the loss or gain of T3SS clusters in different genomic loci. For T3SS proteins, the sequence similarity among orthologs in different genera, especially distantly-related genera, was very low. Therefore, apart from significant sequence similarity (not lower than 30% identity for amino acid sequences), the within-genome synteny information was also considered. Two genes within two different T3SS clusters were also annotated as T3 orthologs if they and their respective two flanking genes had exactly the same gene order, and meanwhile if they shared not lower than 10% amino acid identity. Genes unsatisfied for above criteria could also be considered ‘T3 orthologs’ if they share high similarity in structure (structure orthologs) or function (function ortholog) based on experimental evidence. For structure orthologs, the authors of the original report must have observed and given explanations or discussions for the similarity between two proteins or key domains, and this structure similarity should have similar influence on important protein-protein interaction or protein function with experimental evidence. For function orthologs, experimental evidence is required to support two proteins have similar function. For example, *Salmonella* SptP and *Yersinia* YopE belong to the same ‘T3 ortholog’ cluster because both effectors show GTPase activity and can activate host cytoskeleton proteins [23,24]. Although lacking of apparent sequence similarity, *E. Coli* Map, *Salmonella* SopE and SifA, and *Shigella* IpgB2 all contain a ‘WxxxE’ motif and can mimic guanine nucleotide exchange factors, they were consequently annotated as ‘T3 orthologs’ [25].

## Utility and discussion

The T3DB user interfaces include: (1) browse interface, (2) search interface, (3) download interface and (4) software interface.

In the browse interface (http://biocomputer.bio.cuhk.edu.hk/T3DB/browse), bacterial genera with T3SSs and their phylogenetic relationship between genera was shown in a tree format. Users can select and click any interested genus to browse the genus page. In each genus page, one can skim the basic information about the bacterial genus including host type, interaction type, T3SS number, T3SS names, locations, classes, and the representative strains and their host species in ‘Basic information’ field. Besides, two alternative routes were provided for users to further browse T3SS genes in the genus: by strain and by genus-conserved ortholog cluster. In the “browse by strain” mode, one can click the button of interested bacterial strain within ‘Browse T3SS genes of representative strains’ field, a new interface will be brought out, showing the full list of T3SS genes in this specified strain. Users can learn about the gene distribution and corresponding molecular information in the selected strain. For the “browse by ortholog cluster” mode, in the ‘Browse T3SS gene clusters according to function category’, a general introduction of the number and the function of non-redundant T3SS genes in the genus are provided. Each gene name icon represents a unique genus-conserved ortholog cluster. After selecting an interested gene cluster, a list of T3DB records of all the annotated orthologs in different strains within this genus will appear. The alternative browsing modes are designed to cater for different research interest: either on bacteria or on specific genes. To learn more details about the individual gene in a particular strain, one should click the interested accession record, which leads to the final annotation page for this gene. General information includes gene accession, alias, genomic coordinates, sequence and major function category. The 3D-structure accession (if any) was also linked. The gene was annotated with detailed function, and the references (PMIDs) were included for tracking purpose. Finally, the known intra- or inter-genus T3 orthologs were annotated and within-database links were provided for efficient accession to these orthologs.

T3DB also provides multiple search modes. Users can search an interested T3SS gene through its gene accession, T3ID or gene name. A Blast program was integrated for the users to input query sequence for similarity search (http://biocomputer.bio.cuhk.edu.hk/T3DB/search).

In the download page (http://biocomputer.bio.cuhk.edu.hk/T3DB/download), users may also download gene list and sequences by bacterial genus (or strain) or by function category. Two excel files contain all the well-annotated T3-orthologous clusters and effector-chaperone pairs can be downloaded from this page.

Because T3SS effectors play important roles in host-bacteria interactions, it is of great significance to identify new T3SS effectors. An efficient in silico prediction tool with high sensitivity and high specificity, named BPBAac, was integrated into the database [17]. Users may input a sequence or upload a FASTA file to make predictions. The specificity and sensitivity of the prediction can be freely defined by a user. In addition, links to other T3SS effector prediction softwares were also provided (http://biocomputer.bio.cuhk.edu.hk/T3DB/softwares).

T3SS has received significant research attention in the past years due to its important roles in bacterial pathogenesis and symbiosis. An integrated platform for data storage, data analysis, and knowledge interchange may greatly facilitate T3SS related study in the research community. For example, the function of *Rhizobium* NopA, NopB and NopX proteins have been well studied and annotated (as translocons). These Nop proteins, however, have not yet been identified or studied in other genera, e.g. *Mesorhizobium* and *Bradyrhizobium*. Through searching T3DB, Nop orthologs most likely encoding T3SS translocons were identified in these 3 genera. Furthermore, when comparing the T3SS apparatus genes in the two *Bradyrhizobium* model strains, no NopX ortholog was found in strain *Bradyrhizobium japonicum* USDA 110. According to previous research [26,27], the T3SS in the USDA 110 strain is supposed to be functional. This raises an interesting question as to whether NopX is functionally necessary for T3SSs in all rhizobia. Based on the ortholog clusters, one may also study the phylogenetic relationship among different T3SSs, or the co-evolutionary relationship between different functional categories. The manually-curated high quality effector and chaperone data are useful for feature analysis and evolutionary studies of these special protein groups.

In the future, we plan to extend the T3DB in the following directions: First, to analyze more model strains using bioinformatics and comparative genomics strategies, and to include the T3SS data from non-model strains as well. Second, a transcription regulatory network for different T3SSs will be constructed. Third, protein-protein interaction networks will be integrated to describe the interactions between T3SS proteins and other bacterial proteins, as well as between T3SS effectors and host proteins. We hope that T3DB can make important contribution to T3SS related research in the future.

## Conclusions

We present an integrated T3SS knowledgebase, T3DB, for systematic bacterial T3SS research. T3DB provides comprehensive and well-annotated T3SS-related gene or protein information. T3SS-related genes were annotated according to 4 major categories: apparatus, chaperones, effectors and regulators. All these data were manually annotated, and experimental evidence was provided for each effector, chaperone or transcription regulator. A term, ‘T3 orthologs’, was invented to describe evolutionarily and functionally related T3SS genes. The manually-curated high-quality effector and chaperone data are useful for feature analysis and evolutionary studies of these important proteins. We expect that T3DB will become an important tool for T3SS-related pathogenesis studies for the community.

## Availability and requirements

The link for T3DB is: http://biocomputer.bio.cuhk.edu.hk/T3DB. The database was created and maintained using MySQL. The interactive interfaces were written in PHP. An integrated web server for BPBAac [17] was implemented using PHP and Javascript. The local package for BPBAac was written and implemented with R and Perl.

## Competing interests

We all have no competing interests.

## Authors’ contribution

DG and YW conceived and designed the project. YW and HH collected and annotated the data from literature. YW, MS and QZ maintained the database and implemented web applications. YW wrote the first draft. DG coordinated the study and revised the manuscript. All authors read and approved the final manuscript.

## Funding

This study was funded by a grant from the Institute of Plant Molecular Biology and Agrobiotechnology.
